# Can acoustic measurements predict gender perception in the voice?

**DOI:** 10.1371/journal.pone.0310794

**Published:** 2024-11-14

**Authors:** Diego Henrique da Cruz Martinho, Leonardo Wanderley Lopes, Rodrigo Dornelas, Ana Carolina Constantini

**Affiliations:** 1 Department of Human Development, Universidade Estadual de Campinas—UNICAMP, Campinas, Brazil; 2 Department of Speech and Language Pathology, Federal University of Paraíba–UFPB, João Pessoa, Brazil; 3 Speech Language-Pathology Department, Universidade Federal do Rio de Janeiro–UFRJ, Rio de Janeiro, Brazil; National Taiwan Normal University, TAIWAN

## Abstract

**Purpose:**

To determine if there is an association between vocal gender presentation and the gender and context of the listener.

**Method:**

Quantitative and transversal study. 47 speakers of Brazilian Portuguese of different genders were recorded. Recordings included sustained vowel emission, connected speech, and the expressive recital of a poem. Subsequently, four scripts were used in Praat to extract 16 acoustic measurements related to prosody. Voices underwent Auditory-Perceptual Assessment (APA) of the gender presentation by 236 people [65 speech and language pathologist (SLP) with experience in the area of the voice (SLP), 101 cisgender people (CG), and 70 transgender and non-binary people (TNB)]. Gender presentation was evaluated by visual analogue scale. Agreement analyses were executed among quantitative variables and multiple linear regression models were generated to predict APA, taking the judge context/gender and speaker gender into consideration.

**Results:**

Acoustic analysis revealed that cis and transgender women had higher median fundamental frequency (f_o_) values than other genders. Cisgender women exhibited greater breathiness, while cisgender men showed more vocal quality deviations. In terms of APA, significant differences were observed among judge groups: SLP judged vowel samples differently from other groups, and TNB judged speech samples differently (both p<0.001). The predictive measures for the APA varied based on the sample type, speaker gender, and judge group. For vowel samples, only SLP judges had predictive measures (f_o_ and ABI Jitter) for cisgender speakers. In number counting samples, predictive measures for cisgender speakers included f_o_med and HNR for CG judges, and f_o_med for both SLP and TNB judges. For transgender and non-binary speakers, predictive measures were f_o_med for CG and SLP judges, and f_o_med, CPPs, and ABI for TNB judges. In the poem recital task, predictive measures for cisgender speakers were f_o_med and HNR for both SLP and CG judges, with additional measures of cvint and sr for CG judges, and f_o_med, HNR, cvint, and f_o_peakwidth for TNB judges. For transgender and non-binary speakers, the predictive measures included a wider range of acoustic features such as f_o_med, f_o_sd, sr, fomin, emph, HNR, Shimmer, and f_o_ peakwidth for SLP judges, and f_o_med, f_o_sd, sr, f_o_max, emph, HNR, and Shimmer for CG judges, while TNB judges considered f_o_med, sr, emph, f_o_sd, Shimmer, HNR, Jitter, and f_o_max.

**Conclusions:**

There is an association between the perception of gender presentation in the voice and the gender or context of the listener and the speaker. Transgender and non-binary judges diverged to a higher degree from cisgender and SLP judges. Compared to the evaluation of cisgender speakers, all judge groups used a greater number of acoustic measurements when analyzing the speech of transgender and non-binary individuals in the poem recital samples.

## Introduction

The social construct of gender is a concept that describes how societies attribute different meanings and expectations to people based on their gender identities. Since gender is socially constructed, there are no fixed or universal characteristics. Moreover, gender presentation varies by culture, taking societal characteristics and the current time period into consideration [1, 2].

It is essential to distinguish between three main concepts: gender presentation, gender assignment, and gender identity. Gender presentation refers to the external expression of gender through voice. Gender assignment, on the other hand, refers to the classification of an individual as male or female at birth based on biological characteristics. Gender identity is the internal sense an individual has of their own gender, which may or may not correspond to the gender assignment made at birth [3, 4].

As such, a person is seen by their daily actions, reiterating social norms; and gender presentation involves acting in a way that expresses their gender to the world. A speaker’s performance can affect the interlocutor, influencing social recognition [5].

Starting at birth, people are socialized in accordance with the gender norms specific to their culture. These norms influence behaviors, including the way one communicates and interacts with others in different contexts. Generally speaking, it is through socially shared norms within a culture that people can have expectations about gender presentation. Specifically, people may be expected to fit into one of the binary gender categories (i.e. man or woman) and follow the behavioral patterns associated with these categories [2].

The voice is one of the characteristics by which a speaker’s gender can be perceived [6]. In general, higher-pitched voices are associated with the feminine gender, while lower-pitched voices as associated with the masculine gender [7, 8]. However, the presentation and perception of gender through the voice can be influenced by the context of communication and by the values and preferences of the listener.

Frequency of oscillation (f_o_), measured in Hertz (Hz), is the acoustic correlate of pitch and corresponds to the number of vibrational cycles emitted by the vocal cords during a given interval of time [9]. Traditionally and from the cisgender perspective, f_o_ has been used as one of the main measurements related to the perception of binary genders. Obviously, f_o_ values attributed to the presentation of man and woman voices (in binary terms) can vary among different cultures and languages. For speakers of Brazilian Portuguese, the expectation in cisgendered terms is that men present an f_o_ between 80Hz and 150Hz and women between 150Hz and 250Hz [10].

While many cultures have a predominance of f_o_ values similar to those found in Brazil, such as American English with expected averages of 118Hz for men and 208Hz for women [11], the average f_o_ values for some languages such as Wu Chinese are nearly equivalent for man and woman speakers [12]. This also occurs in Danish, in which the difference in f_o_ between genders is relatively small [13], in contrast with Russian, in which differences are relatively larger [14]. In different cultures and social contexts, expectations in relation to the voice can differ [2, 7]. For example, in Yoruba culture, it is more appropriate for women to have firmer and more commanding voices [2], while in Brazilian or occidental culture, a softer and more melodic voice can be valued [7]. Cultural norms mold and direct the production and perception of the voice in function of gender. As such, sociocultural variables and the preferences and values of listeners influence the perception of gender meaning that f_o_ values constitute only one of the variables related to this perception [15–18].

It is common for transgender and genderfluid people to search for specialized treatments to modify their voices due to feeling that their voices do not represent them [8, 19]. In these cases, the job of a speech and language pathologist involves respecting the self-designation of the person and autonomy in constructing therapeutic objectives. Interventions can include adjustments in resonance [15, 17, 18, 20–24], softening emission with an increase in breathiness [17, 25], improving articulatory precision [15, 18], and even phono-articulatory adjustments such as modifying the position of the tongue [26].

Besides vocal characteristics, some studies discuss behavioral factors related to gender with an emphasis on pragmatic elements of discourse [18, 21, 22] such as prosody [17, 18, 21–24] and other aspects of non-verbal communication such as body language and the use of gestures [21–23]. The inclusion of these characteristics in phonoaudiological therapy reinforce that the perception of gender in the voice is multifactorial and, therefore, these same characteristics may be related to different acoustic measurements correlated, or not, with the aforementioned aspects.

Current scientific productions regarding gender presentation has focused predominantly on the analysis and suitability of the voice with a foundation in binary stereotypes of femininity and masculinity [15, 17, 18, 20–26]. However, a considerable gap resides in the scarcity of studies that consider and understand the different perceptions of gender to beyond the cisgender viewpoint. This gap limits the comprehension of individual experiences and necessities which transcend binary categories here.

A previous study using the same samples as this research highlighted that gender perception in the voice can vary depending on the judge group, indicating a significant influence of the evaluator’s context on judgments [27]. This finding evidence the need for the present study and raises the hypothesis that the evaluator’s background may also lead to differences in the evaluation of voices, whether from cisgender or transgender individuals. In other words, it explores how each judge group responds when confronted with gender diversity in voice.

In sociophonetics, the study of gender perception in the speaker’s voice as a continuous variable is well-established. Previous research has shown that distinct patterns of phonetic variation allow listeners to make inferences about sexual orientation or perceptions of masculinity and femininity, highlighting that these perceptual parameters are interrelated but distinct [28]. Similarly, variations in pitch and specific phonetic characteristics can be interpreted differently across languages; for instance, higher pitches are often associated with more effeminate voices. However, the perception of these cues can vary significantly depending on the listener’s linguistic and cultural context [29]. Thus, analyzing gender presentation as a continuum is an extension of a robust body of research exploring the subtleties of how vocal characteristics convey gender and sexual orientation.

Furthermore, in Speech-Language Pathology, some studies have utilized analogical-visual scales to assess how gender is perceived in the voice, emphasizing the importance of considering multiple perspectives beyond traditional binary models [27, 30–33]. Factors such as sexual orientation, gender identity, and listeners’ familiarity with the LGBTQIAPN+ community can influence their perceptions of gender in the voice [34, 35]. In the United States, for example, the influence of linguistic context and listeners’ gender identity on identifying the gender of transgender speakers seems relatively limited, suggesting that gender perception is more fluid and continuous rather than strictly binary [35].

This study stems from the premise that other acoustic measurements, besides f_o_, contribute to the perception of the presentation of gender in the voice and that this perception is variable as a function of the context of the listener. This study innovates by utilizing gender as a continuous, non-categorical variable in Brazilian speakers and by considering the perspective of people with different gender identities. The presented approach can contribute to a deeper understanding of the complex interaction between the voice, the perception of gender, and the context of the listener, recognizing that the variability of life experiences and interactions with femininities and masculinities has the potential to offer a broader and more sensitive viewpoint to orient more inclusive and suitable practices. As such, the objective of this study was to determine if there is an association between the perception of gender presentation in the voice and the gender and context of the listener, as well as to analyze the acoustic measurements that can predict the perception of gender presentation through the voice.

## Method

Quantitative, observational, transversal, prospective study submitted to and approved by the Committee of Ethics in Research (Comitê de Ética em Pesquisa), file number 4.730.175. Data collection was carried out following the same methodology as Martinho & Constantini (2024) [27] and was performed between August 2021 and January 2022 in a school-clinic at a university in the city of Campinas, São Paulo, Brazil. All participants signed the informed consent form in writing.

### Selection and recording of participants

Participants were recruited by social media and by e-mail through invitations sent by Lesbian, Gay, Bisexual, Transgender, Queer/Questioning, Intersex, Asexual/Aromantic/Agender, Pansexual/Polysexual, Non-Binary, and more (LGBTQIAPN+) reference centers. The snowball sampling methodology was adopted. This characterizes a non-probabilistic convenience sample. The study was conducted in 2 stages and the first stage involved the recording the voices of 47 speakers of Brazilian Portuguese: 11 cisgender women, 11 transgender/travesti woman, 11 cisgender men, 7 transgender men, and 7 non-binary people. The participants that had their voices recorded were between the ages of 18 and 47 (average 25.94; median 24). In gender divisions, the group of transgender women included transexual women and travestis. As a methodological choice, these people were chosen to be grouped, since both identities perform gender roles that express aspects of femininity and, in Brazil, the identity of a travesti can be adopted through a political manifestation that goes back to the beginning of the discussion of transgenderism in the country.

Exclusion criteria were: the presence of health problems that could affect vocal quality on the day of data collection (such as flu, cold, or airway infections, as self-reported by the participant); continuous use of medication that could interfere with vocal production (except for hormonal therapy); and the use of tobacco. In addition, the participant could not have reported any vocal health issues on the day of collection.

**Fig 1:**
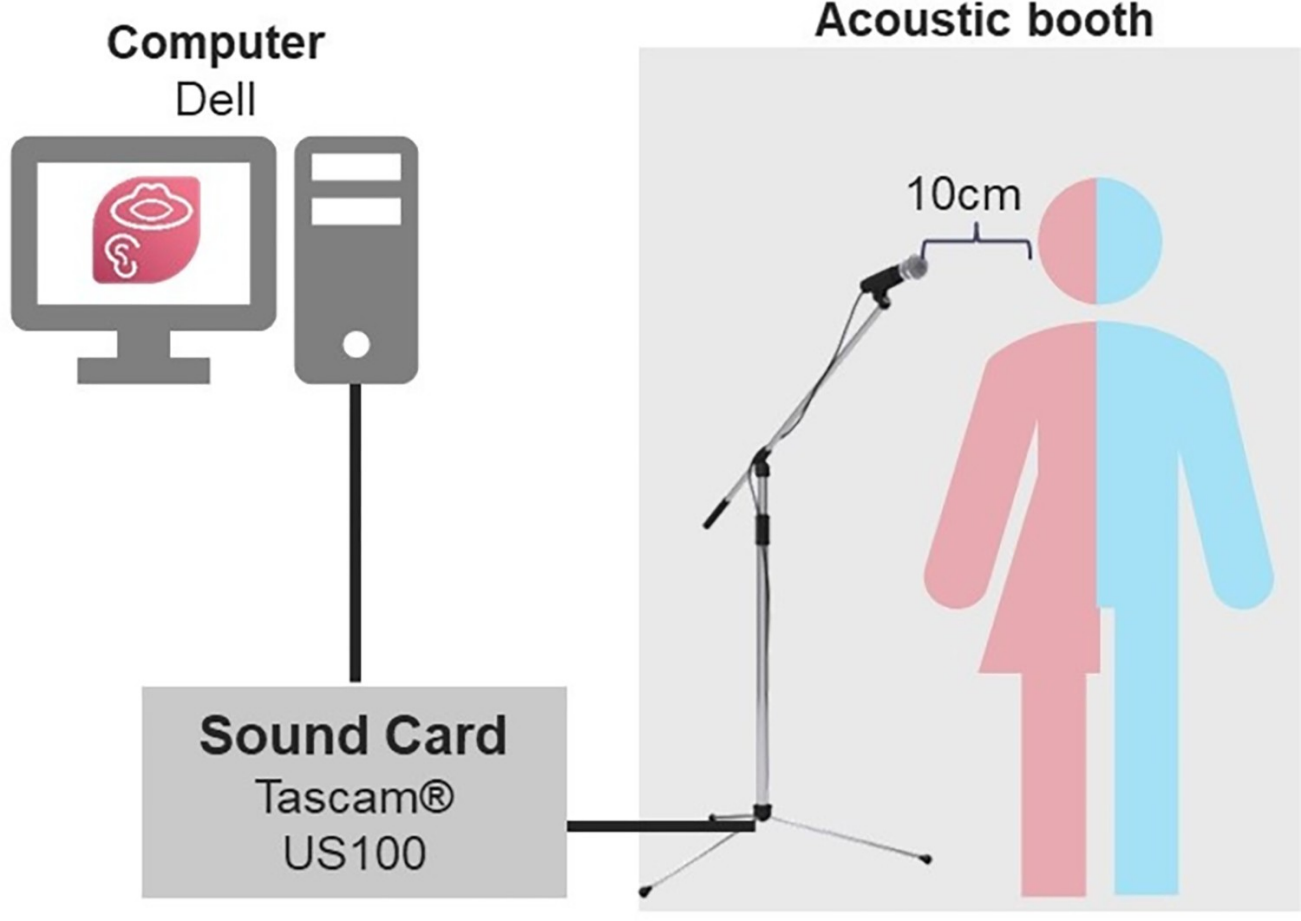
Recording environment.

After receiving instructions about the procedures involved in the research, the participant was directed to an acoustically controlled recording area with background noise levels below 50dB SPL. Recordings were performed with the participant standing inside an acoustic cabinet, using a unidirectional microphone placed 10 centimeters from the mouth. For this procedure, a Dell desktop computer and Shure® microphone (model SM58) coupled with a Tascam® sound card (model US100) were used. Speakers were recorded directly through Praat software (version 6.2.14) [36] with a sampling rate of 44kHz. The recording environment is depicted in Fig 1.

The recording procedure consisted of the emission of three sustained [a] vowels, chosen for its more neutral vocal tract configuration and for permitting the evaluation of gender without communicative characteristic influences; the emission of an ascending-descending glissando using the [a] vowel, to identify vocal extension; connected speech (counting numbers from one to ten); and the recital of the poem “O amor bate na Aorta” written by the Brazilian author Carlos Drummond de Andrade [37]. For this last task, the participant was instructed to recite the poem expressively, with the command “recite the following poem with emotion”. These tasks are necessary to evaluate voice dynamics by way of acoustic analysis and Auditory-Perceptual Assessment.

The selection of the aforementioned tasks was strategic to encompass different aspects of vocal production, considering whether or not there is interference from communicative aspects.

### Extraction of acoustic measurements

The following were selected for extraction and analysis: acoustic measurements related to f_o_, measurements of noise perturbations related to breathiness and strain, as well as measurements related to articulation and prosody. Acoustic measurements selected for this study can be found in Table 1.

**Table 1:** Acoustic measurements chosen for extraction and analysis.

Measurement	Description
**f**_**o**_ **med**	Median frequency of oscillation.
**f**_**o**_ **min**	Minimum f_o_ marked in the sample.
**f**_**o**_ **max**	Maximum f_o_ marked in the sample.
**f**_**o**_ **sd**	Standard deviation of f_o_, measuring the variability of the frequency of oscillation in the sample, related to the liveliness of speech. Larger values indicate a larger number of accents and emphases [38].
**f**_**o**_ **peakwidth**	Average bandwidth of the f_o_ peaks, a measurement of how peaks are formed, impacting perception of charisma or persuasion [39].
**Jitter (%)**	Variations in the periodicity of the wave, cycle-to-cycle, revealing possible irregularities in the vibrations of the vocal cords. There may be a relationship between jitter and emotional activation [40].
**Shimmer (dB)**	Cycle-to-cycle variations in the amplitude of the waveform.
**emph**	Spectral emphasis in dB, increasing in value with increasing vocal effort [41].
**cvint**	Variation coefficient of total intensity, equal to the standard deviation of the global intensity divided by the average of the global intensity.
**HNR (dB)**	Harmonic-to-noise ratio that separates the audio into two parts. Low values indicate more breathiness [42].
**H1-H2 (dB)**	An indirect measurement of the relative duration of the open phase of the vocal cords. In breathier voices, the amplitude of the first harmonic is relatively high in comparison with the following harmonics [43].
**CPPs (dB)**	Smoothed cepstral peak prominence, representing the separation of vocal processes and breathiness and strain, with more strained voices having higher CPPs values [44].
**AVQI**	Acoustic vocal quality index. A combination of six acoustic measurements to provide a single score from 0 to 10 points [45].
**ABI**	Acoustic breathiness index. A combination of nine acoustic measurements to provide a single score from 0 to 10 points [46].
**Sr**	Speech rate, measured by the number of vowel-vowel units per second, including pauses.
**Ar**	Articulation rate, measured by the number of vowel-vowel units, excluding pauses.

Acoustic measurements were extracted from the sustained [a] vowel samples, connected speech, and poem recital using Praat [36] software and four scripts, detailed below.

The choice of a wide range of acoustic measures is justified by the importance of prosodic, acoustic, and communicative aspects in gender perception. The fundamental frequency (F0) plays a crucial role in this context [8], making the inclusion of various descriptors of this measure essential for analysis. Additionally, noise-related characteristics were considered to assess vocal quality and stability. Prosodic aspects are also crucial as they reflect the dynamics and expressiveness of speech, which are significant elements in expressing gender identity. Measures of vocal quality were integrated to evaluate timbre and overall clarity of the voice, complementing the analysis.

The literature reveals that acoustic measures vary significantly according to the speaker’s gender. For example, phonetic differences observed between cisgender individuals include higher formant frequencies and slower speech rates in women [47]. Measures such as Cepstral Peak Prominence (CPP) and the difference between the first two harmonics (H1-H2) are useful for analyzing vocal quality, showing that voices perceived as feminine tend to be breathier [48]. Additionally, research indicates that cisgender women produce vowels with longer duration and higher formant frequencies when compared to cisgender men [49].

#### ABI and AVQI

The AVQI and ABI are multiparametric indices where AVQI evaluates overall vocal quality and ABI quantifies breathiness present in the voice. Both of the indices consider connected speech and the sustained [a] vowel to give a single final score, from zero to ten, in which zero corresponds to the absence of deviations and ten corresponds to extreme vocal deviation [50].

The AVQI (version 03.01) takes six acoustic measurements into consideration for its score: smoothed cepstral peak prominence (CPPs), harmonics-to-noise ratio (HNR), local shimmer (SL), local shimmer dB (SLdB), general slope of the long-term average spectrum (Slope), and tilt of the regression line through the long-term average spectrum (Tilt), according to the mathematical formula below [45].


AVQI=[4,152−(0,177×CPPs)–(0,006×HNR)–(0,037×Shimmer)+(0,941×ShimmerdB)+(0,01×Slope)+(0,093×Tilt)]×2,8902.

The ABI considers nine acoustic measurements to calculate its score: smoothed cepstral peak prominence (CPPs), jitter, glottal-to-noise excitation ratio at a maximum frequency of 4500 Hz (GNEmax-4500Hz), high-frequency noise value between 0-6kHz and 6-10kHz (Hfno-6000Hz), Dejonckere and Lebacq harmonic-to-noise ratio (HNR-D), difference between the first and second harmonic (H1-H2), shimmer dB, shimmer, and period standard deviation (PSD), according to the mathematical formula below [46].


ABI=(5,0447730916–[0,172×CPPs]–[0,193×Jitter]–[1,283*GNEmax−4500Hz]–[0,396×Hfno−6000Hz] + [0,01×HNR−D] + [0,017×H1−H2] + [1,473×Shim−dB]–[0,088×Shim]–[68,295×PSD])×2,9257400394

The choice to employ AVQI and ABI in the analysis of the cohort composed of normal voices was motivated by the need for a comprehensive and objective assessment of vocal quality, these are current and robust measures that have been studied in various languages and populations [45, 46, 50]. This consideration arises from the understanding that gender perception in the voice extends beyond traditional measures of oscillatory frequency. This approach allows for an exploration of whether vocal quality and breathiness measures can impact how gender is perceived.

Two files were imported into Praat [36] to extract the ABI and AVQI indices, the connected speech file (counting numbers) and a 3-second sustained [a] vowel file. The files were handled following orientations provided by the authors of the script [45, 46]. The results of the individual acoustic measurements that compose the indices and index scores were extracted and tabulated.

#### Prosody descriptor and mark-pauses

We used the Prosody Descriptor script [51] for the extraction of prosodic-acoustic measurements, performed separately for each type of vocal sample (connected speech and poem recital).

**Fig 2:**
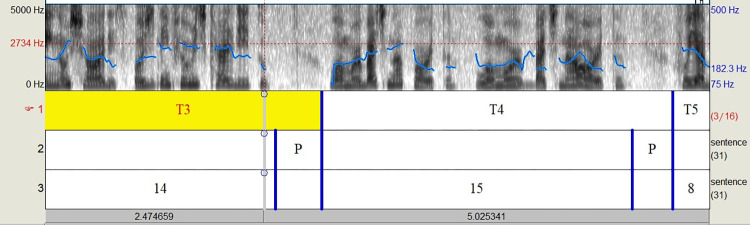
Segmentation in Praat utilizing a three-layer TextGrid. Layer 1 –speech segments; Layer 2- silent pauses; Layer 3- segment of speech with the number of phonetic syllables in that segment.

Descriptor requires that the audio files be labeled in Praat [36], identifying the pauses and number of phonetic syllables in each segment of speech. To standardize pause segmentation, the Mark-pauses script [52] was used. The script identifies silent pauses and performs automatic segmentation. After checking, the speech segments and phonetic syllables were marked on the corresponding lines. An example of segmentation can be seen in Fig 2, below. After executing, the Prosody Descriptor script generated a report in text file format with all the extracted acoustic measurements, which were organized into tables.

### Auditory-Perceptual Assessment

In the second stage of the study, APA of gender presentation in voices was carried out using the previously recorded files from stage 1. Gender presentation in the voices was judged by 236 people: 65 speech and language pathologists with experience in the area of voice (SLP), 101 cisgender people (CG), and 70 transgender and non-binary people (TNB). The total group of judges were between the ages of 18 and 68 years (average 31.84 years; median 27 years).

The selection of individuals who conducted the judgments was guided by the need to understand and to explore how transgender individuals perceive gender in the voice, as well as whether speech-language pathologists working in the field of voice have a perception similar to cisgender individuals. This relates to our hypothesis that people perceive gender differently depending on their context and background.

Inclusion criteria for this stage were: be a native speaker of Brazilian Portuguese and be 18 years of age or older. A SLP needed to have been practicing for at least six months in the area of the voice. All SLP participants were cisgender and the difference between this group and CG was their specialty in the voice. Exclusion criteria were: participation in the recording stage or the presentation of any auditory issues.

For this stage, two questionnaires were created using the SurveyMonkey® platform: one questionnaire with segments of spoken voice and the other questionnaire containing the sustained [a] vowel. Judges did not have information about the speaker that provided this voice sample, and the presentation of vocal samples in both questionnaires was random, so that each judge evaluated the voices in a different order.

For the sample composition available in the questionnaire, voices were segmented in Praat so that the following samples could be evaluated: I–complete sample of the central [a] vowel (second of three requested repetitions); II–sample with connected speech (counting from one to ten) and the first verse of the poem. Each segment was presented in a different questionnaire with each vowel sample lasting 5 seconds and the speech samples lasting 15 seconds each. In addition, nine (20%) of the samples in each task were randomly repeated to calculate evaluation consistency of the judge. For the confidence calculation, an F-test was used considering a p-value cutoff of 0.05.

**Fig 3:**
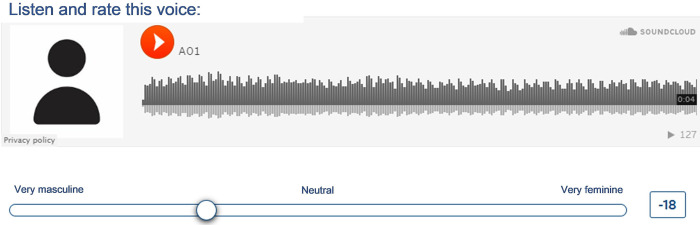
Scale for judging gender presentation in the voice.

Judges evaluated gender presentation in the voice using a visual analogue scale with 101 points allowing the evaluation of gender on a continuous scale with the possibilities of this scale ranging from very masculine voice (attributed score of -50) to the far left, to very feminine (attributed score of 50) to the far right, as shown in Fig 3. The center point of the scale (attributed score of zero) represented neutral voices, those which the judges could not classify the gender.

#### Analysis of the results

**Fig 4:**
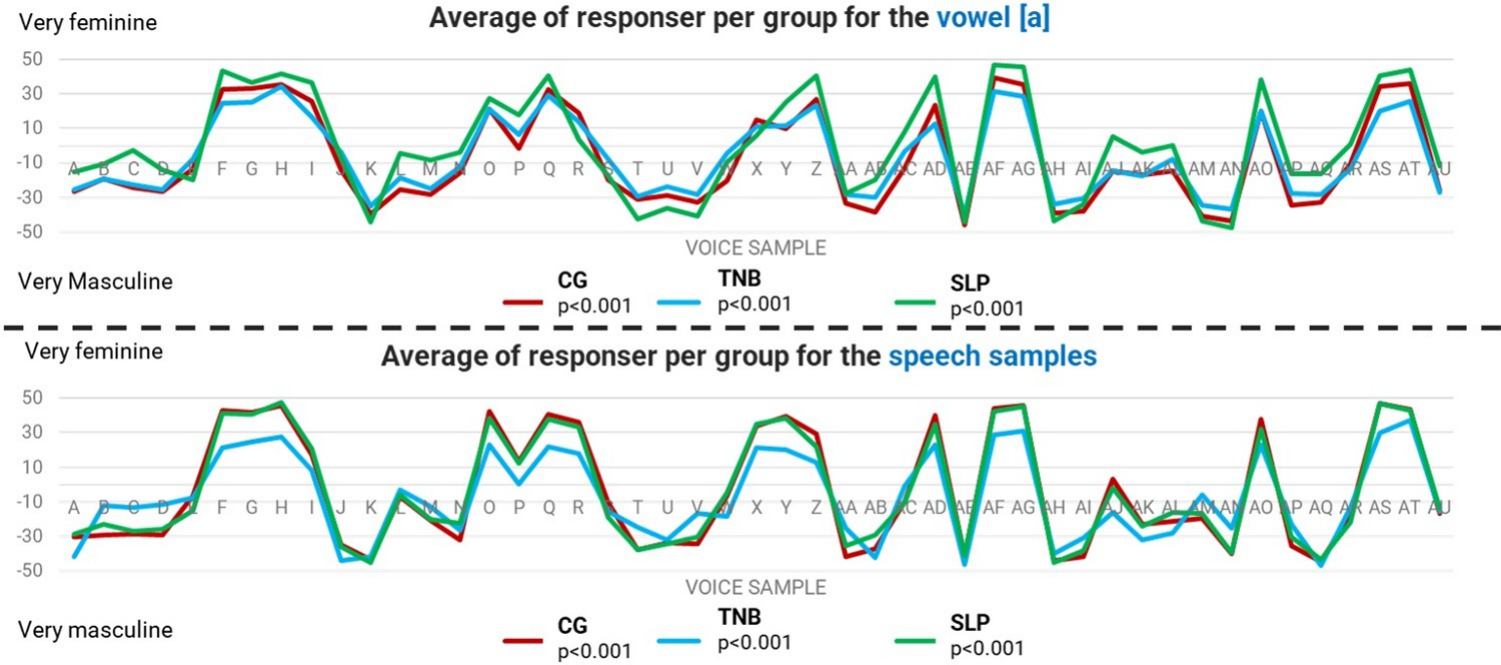
Summary of the research procedures.

The data were grouped into tables and analyzed in a descriptive and inferential manner utilizing SPSS 25.0 software. A significance level of 5% was considered for inferential analyses. The inferential agreement analysis between quantitative variables was performed using the Intraclass Correlation Coefficient. Multiple linear regression models were used to predict dependent variables. Independent variable selection was performed using the stepwise method. The Fig 4 summarizes all the research procedures.

## Results

### Acoustic analysis

This section presents the descriptive analysis of extracted acoustic measurements from the different speech tasks. The ABI indicated that eight (17.02%) participants obtained scores above the cutoff, suggesting that they presented a degree of breathiness above what was expected. The same occurred for the AVQI, in which 17 (36.17%) participants were above the cut off, indicating possible deviations in vocal quality. Table 2 shows the medians of the extracted acoustic measurements in accordance with the analyzed speech task and the gender of the speaker. Note that cis and trans women have a median f_o_ higher in relation to other genders. Cis women showed the highest levels of breathiness in the voice according to the ABI score followed by non-binary people and trans women. Cis men expressed the highest indices of vocal quality alteration according to the AVQI score, followed by trans men and non-binary people.

**Table 2:** Extracted acoustic measurements from the different speech tasks considering the gender of the speakers.

Speech Task	Acoustic measurement	Cis Woman	Cis Man	Non-binary	Trans Woman	Trans Man
		Median	Median	Median	Median	Median
**[a] vowel**	**Modal f**_**o**_ **(Hz)**	213.90	124.17	135.26	156.09	134.08
	**Minimum f**_**o**_ **(Hz)**	161.76	104.29	103.21	118.38	127.14
	**Maximum f**_**o**_ **(Hz)**	417.06	374.07	325.09	406.75	237.12
**Vowel and numbers**	**CPPs (dB)**	14.79	15.30	14.42	15.21	16.13
	**Jitter (%)**	0.98	1.34	1.35	1.40	1.28
	**H1-H2 (dB)**	5.11	-1.04	2.20	2.31	0.79
	**Shimmer (dB)**	0.39	0.53	0.49	0.42	0.44
	**Shimmer (%)**	3.74	4.36	4.80	3.51	4.21
	**HNR (dB)**	20.60	21.13	20.52	18.80	20.69
	**ABI Score**	2.79	1.60	2.57	1.97	1.72
	**AVQI Score**	1.06	1.38	1.13	1.09	1.19
**Numbers**	f_o_**med (Hz)**	209.00	120.00	120.00	136.00	135.00
	f_o_**sd (Hz)**	21.45	16.80	13.23	18.80	15.40
	f_o_**min (Hz)**	154.00	101.00	109.00	115.00	114.00
	f_o_**max (Hz)**	265.00	178.00	163.00	205.00	188.00
	f_o_**peakwidth (Hz)**	34.30	22.20	44.30	18.90	34.00
	**emph (dB)**	1.50	1.30	2.00	1.20	1.20
	**cvint (dB)**	33.00	35.00	36.00	37.00	34.00
	**HNR (dB)**	16.00	11.70	11.00	14.20	12.60
	**shimmer (dB)**	4.50	5.50	5.90	5.50	5.50
	**jitter (%)**	1.40	1.50	1.50	1.70	1.70

f_o_−frequency of oscillation; CPPs–smoothed cepstral peak prominence; H1-H2 –difference of the first two harmonics; HNR–harmonic-to-noise ratio; ABI–acoustic breathiness index; AVQI–acoustic voice quality index; f_o_med–f_o_ median; f_o_sd–standard deviation of f_o_; f_o_min–minimum f_o_; f_o_max–maximum f_o_; f_o_peakwidth–average width of f_o_ peaks; emph–spectral emphasis; cvint–coeffient of variation of vocal intensity

### Auditory-Perceptual Assessment

Testing the inter-evaluator consistency resulted in statistical significance in all groups (p<0.001); however, intra-evaluator consistency measured by F-test resulted in 79 (32.3%) of the judges having significance above the p-value cutoff for the vowel judgement and nine (3.7%) for speech. These data indicate that the judges had more difficulties judging gender in the voice using only the sustained vowel sample. As such, only judges that presented low consistency (p>0.05 in the F-test) with the speech task were excluded since we considered that the speech sample analysis offered a larger number of acoustic cues to provide a reliable analysis. All of the excluded judges (n = 9) were cisgender women, two from the CG group and seven from the SLP group.

The means, medians, and standard deviations of the evaluation of gender presentation according to judges can be found in Table 3, grouped by speaker gender. Only cisgender women had their gender presentation perceived as feminine (positive mean and median values) and cisgender men had their vocal gender presentation perceived as more masculine with values close to -50. Non-binary people, trans women and/or travestis and trans men were closer to the neutral range, with values near zero.

**Table 3:** Mean, median, and standard deviation of scores obtained from the APA stage, grouped by speaker gender and judge group.

		CG		SLP		TNB	
Gender		VOWEL	SPEECH	VOWEL	SPEECH	VOWEL	SPEECH
**CM**	**MEAN**	-32.60	-37.68	-28.89	-35.87	-28.27	-30.39
	**MEDIAN**	-32.91	-37.70	-33.72	-35.08	-27.90	-30.84
	**SD**	4.96	5.69	14.27	6.55	3.87	11.07
**TM**	**MEAN**	-2.60	3.88	9.83	0.36	0.06	-2.75
	**MEDIAN**	-10.53	-6.37	7.98	-10.28	-3.01	-0.71
	**SD**	15.84	24.01	20.00	24.01	14.58	17.27
**CW**	**MEAN**	29.85	42.01	37.17	39.73	24.36	25.56
	**MEDIAN**	32.77	42.69	40.60	40.94	25.40	23.14
	**SD**	7.34	3.00	12.23	4.84	6.75	5.41
**TW**	**MEAN**	-13.65	-11.23	-2.59	-9.62	-9.45	-17.02
	**MEDIAN**	-15.57	-16.55	-7.18	-15.91	-7.87	-16.14
	**SD**	18.58	22.26	15.43	21.17	14.74	19.28
**NB**	**MEAN**	-23.69	-17.04	-17.52	-14.79	-21.85	-10.47
	**MEDIAN**	-26.32	-28.95	-13.98	-22.72	-25.31	-11.71
	**SD**	27.62	30.56	31.25	30.00	20.29	22.97

CM: Cisgender man; TM: Trans man; CW: Cisgender woman; TW: Trans woman and/or *travesti*; NB: Non-binary person; CG: Cisgender group; SLP: Speech and language pathologists with experience in the voice; TNB: Trans and non-binary group.

**Fig 5:**
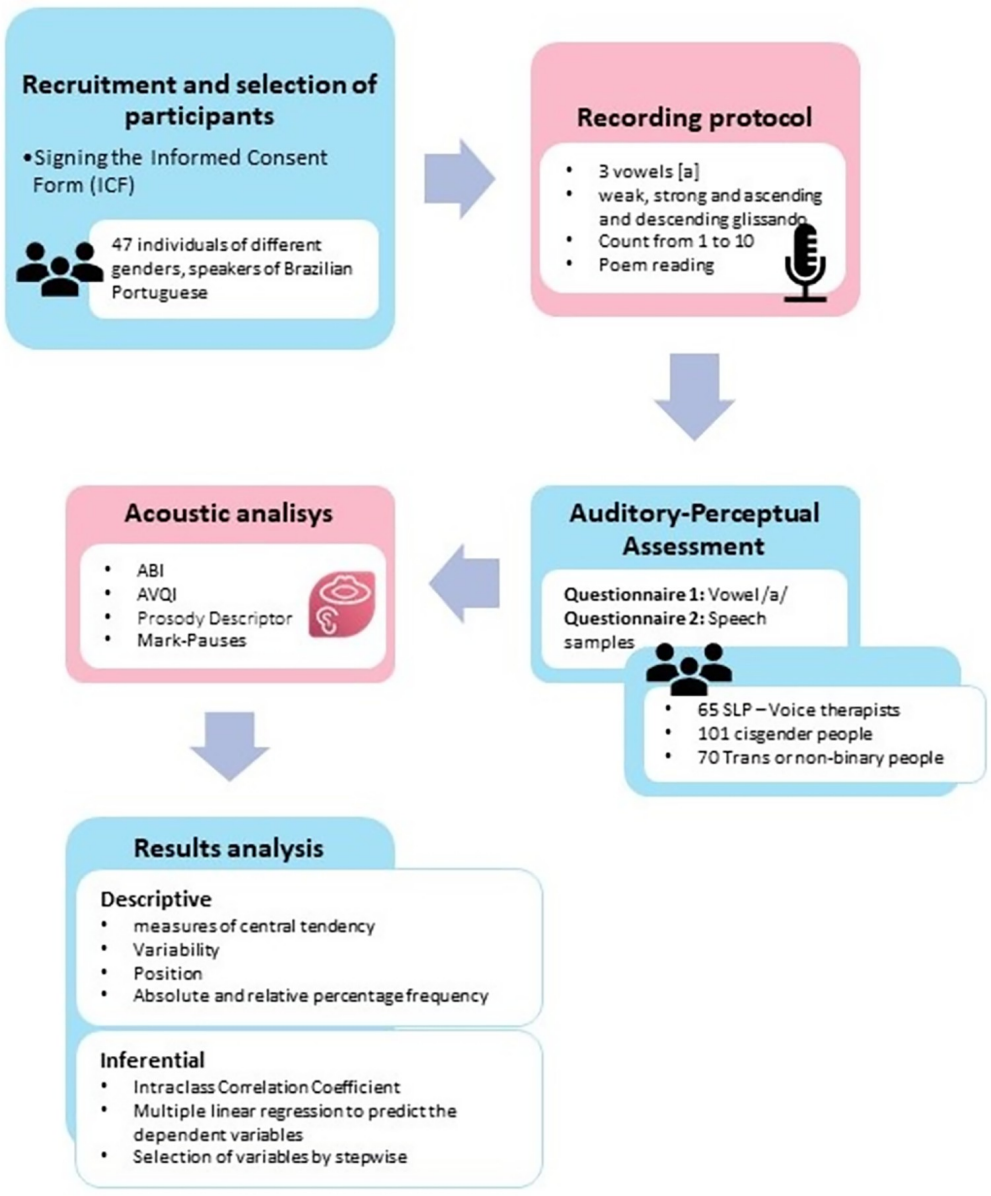
Averages of the perception of gender in each vocal sample group in the vowel and speech tasks. CG: Group of cisgender people; SLP: Group of speech and language pathologists with experience in the voice; TNB: Group with trans and non-binary people.

Fig 5 demonstrates a visual description of the average APA of gender presentation in the voice for each vocal sample in each judge group for the sustained vowel and speech tasks. The highest level of disagreement can be seen in the SLP group for the sustained [a] vowel task and in the TNB group for the speech tasks. Disagreements in judgement are indicated by divergences from overlapping line.

#### Predictive analysis of Auditory-Perceptual Assessment

Multiple linear regression models were generated with the following objectives:

- Calculate acoustic measurements that are predict Auditory-Perceptual Assessment of the gender of the speaker for each group of listeners;
- Determine how each group of listeners evaluates speaker gender.

#### Vowel samples

Two multiple linear regression models were generated for each group of judges to determine if the acoustic measurements of vowel analysis can predict APA for the vowel samples in CG and TNB groups.

For the CG and TBN judge groups, none of the extracted acoustic measurements from the [a] vowel demonstrated the capacity to predict Auditory-Perceptual Assessment by these groups for these vowel samples. For judges in the SLP group, a statistically significant model was generated for APA of the vowel of cisgender speakers (p<0.001), seen in Table 4.

**Table 4:** Predictive analysis of Auditory-Perceptual Assessment in vowel sample from cisgender speakers by SLP judges.

	Unstandardized coefficients		Standardized coefficients	t	p-value
	B	Standard Error	Beta		
(Constant)	-188.078	27.877		-6.747	<0.001
[a] vowel f_o_	0.882	0.081	1.131	10.833	<0.001
ABI Jitter (%)	37.535	13.498	0.290	2.781	0.012

Stepwise multiple linear regression

ABI Jitter–Jitter measurement extracted from the ABI script

#### Speech samples

Four multiple linear regression models were generated for each group of judges to determine if the independent variables, the extracted acoustic measurements from connected speech (number reading or poem recital), are capable of predicting APA from the speech sample in two different speaker groups (cisgender speakers or trans and non-binary speakers), the dependent variable.

The acoustic measurements extracted from number counting samples that were predictive of APA (p<0.001) in the three judge groups can be found for the evaluation of cisgender speakers in Table 5 and trans and non-binary speakers in Table 6. Acoustic measurements extracted from the poem recital samples can be found for cisgender speakers in Table 7 and trans and non-binary speakers in Table 8.

**Table 5:** Predictive analysis of Auditory-Perceptual Assessment of number counting samples from cisgender speakers using extracted acoustic measurements.

	Unstandardized coefficients		Standardized coefficients	t	p-value
	B	Standard Error	Beta		
**CG**					
(Constant)	-196.981	26.548		-7.420	<0.001
f_o_med	0.923	0.057	0.985	16.228	<0.001
AVQI HNR (dB)	2.597	1.145	0.138	2.268	0.035
**SLP**					
(Constant)	-134.930	8.856		-15.236	<0.001
f_o_med	0.860	0.054	0.963	16.003	<0.001
**TNB**					
(Constant)	-104.409	8.612		-12.124	<0.001
f_o_med	0.641	0.052	0.939	12.264	<0.001

Stepwise multiple linear regression

f_o_med–Median f_o_; AVQI HNR–Harmonic-to-noise ratio extracted from the AVQI script

**Table 6:** Predictive analysis of Auditory-Perceptual Assessment of number counting samples from trans and non-binary speakers using extracted acoustic measurements.

	Unstandardized coefficients		Standardized coefficients	t	p-value
	B	Standard Error	Beta		
**CG**					
(Constant)	-103.174	12.104		-8.524	<0.001
f_o_med	0.675	0.084	0.858	8.010	<0.001
**SLP**					
(Constant)	-102.221	9.926		-10.298	<0.001
f_o_med	0.670	0.069	0.897	9.706	<0.001
**TNB**					
(Constant)	-286.692	64.452		-4.448	<0.001
f_o_med	0.225	0.110	0.366	2.046	0.053
CPPs (dB)	13.308	3.962	0.935	3.359	0.003
ABI Score	19.830	7.916	0.723	2.505	0.021

Stepwise multiple linear regression

f_o_med–Median f_o_; CPPs–Smoothed cepstral peak prominence; ABI Score–score from the acoustic breathiness index

**Table 7:** Predictive analysis of Auditory-Perceptual Assessment of poem recital samples from cisgender speakers using extracted acoustic measurements.

	Unstandardized coefficients		Standardized coefficients	t	p-value
	B	Standard Error	Beta		
**CG**					
(Constant)	-100.456	12.789		-7.855	<0.001
f_o_med	0.697	0.024	0.838	28.621	<0.001
HNR	1.801	0.518	0.102	3.477	0.001
Cvint	-1.053	0.367	-0.068	-2.865	0.004
Sr	-3.565	1.562	-0.053	-2.282	0.023
**SLP**					
(Constant)	-133.638	4.504		-29.672	<0.001
f_o_med	0.666	0.022	0.844	30.095	<0.001
HNR	1.954	0.472	0.116	4.143	<0.001
**TNB**					
(Constant)	-87.981	7.617		-11.551	<0.001
f_o_med	0.497	0.019	0.824	26.387	<0.001
HNR	1.331	0.398	0.103	3.346	0.001
Cvint	-0.791	0.262	-0.070	-3.023	0.003
f_o_peakwidth	0.122	0.052	0.053	2.341	0.020

Stepwise multiple linear regression

f_o_med–Median f_o_; HNR–harmonic-to-noise ratio; cvint–Coefficient of variation of total intensity; sr–Speech rate; f_o_peakwidth–Average bandwitch of f_o_ peaks.

**Table 8:** Predictive analysis of Auditory-Perceptual Assessment of poem recital samples from trans and non-binary speakers using extracted acoustic measurements.

	Unstandardized coefficients		Standardized coefficients	t	p-value
	B	Standard Error	Beta		
**CG**					
(Constant)	-165.089	7.587		-21.760	<0.001
f_o_med	0.664	0.032	0.818	21.059	<0.001
f_o_sd	-0.827	0.108	-0.282	-7.629	<0.001
Sr	2.354	0.646	0.083	3.646	<0.001
f_o_max	0.080	0.027	0.153	2.998	0.003
Emph	6.251	1.213	0.123	5.154	<0.001
HNR	2.042	0.347	0.197	5.893	<0.001
Shimmer	2.077	0.430	0.143	4.829	<0.001
**SLP**					
(Constant)	-164.371	7.564		-21.731	<0.001
f_o_med	0.619	0.036	0.787	17.318	<0.001
f_o_sd	-0.394	0.084	-0.139	-4.701	<0.001
Sr	2.425	0.695	0.088	3.492	0.001
f_o_min	0.088	0.039	0.085	2.272	0.023
Emph	5.580	1.197	0.113	4.661	<0.001
HNR	2.274	0.343	0.226	6.630	<0.001
Shimmer	2.237	0.432	0.159	5.181	<0.001
f_o_peakwidth	0.064	0.030	0.049	2.123	0.034
**TNB**					
(Constant)	-151.904	8.732		-17.396	<0.001
f_o_med	0.454	0.032	0.681	14.354	<0.001
Sr	2.410	0.713	0.103	3.381	0.001
Emph	8.897	1.270	0.213	7.004	<0.001
f_o_sd	-0.540	0.109	-0.225	-4.970	<0.001
Shimmer	2.143	0.462	0.179	4.641	<0.001
HNR	2.433	0.384	0.286	6.341	<0.001
Jitter	4.003	1.381	0.106	2.898	0.004
f_o_max	0.054	0.027	0.125	1.997	0.046

Stepwise multiple linear regression

f_o_med–Median f_o_; f_o_sd–Standard deviation of f_o_; sr–speech rate; f_o_max–Maximum f_o_; emph–Spectral emphasis; HNR–harmonic-to-noise ratio; f_o_min–Minimum f_o_; f_o_peakwidth–Average bandwidth of f_o_ peaks

Table 9 presents a summary of the predictive measures in each speech task according to the judge and speaker groups.

**Table 9:** Summary of predictive measures for each listener and speaker group in vowel, speech, and poem recital.

Sample	Vowel		Number Counting		Poem Recital	
Speaker	Cisgender	Transgender and Non-binary	Cisgender	Transgender and Non-binary	Cisgender	Transgender and Non-binary
**SLP**	f_o_, ABI	-	f_o_med	f_o_med	f_o_med, HNR	f_o_med, fosd, sr, f_o_min, emph, HNR, Shimmer, fopeakwidth
**CG**	-	-	f_o_med, HNR	f_o_med	f_o_med, HNR, cvint, sr	f_o_med, fosd, sr, f_o_max, emph, HNR, Shimmer
**TNB**	-	-	f_o_med	f_o_med, CPPs, ABI	f_o_med, HNR, cvint, f_o_peakwidth	f_o_med, sr, emph, f_o_sd, Shimmer, HNR, Jitter, f_o_max

ABI–score from the acoustic breathiness index; f_o_med–Median f_o_; f_o_sd–Standard deviation of f_o_; CPPs–Smoothed cepstral peak prominence; HNR–harmonic-to-noise ratio; sr–speech rate; f_o_min–Minimum; f_o_max–Maximum f_o_; emph–Spectral emphasis; f_o_peakwidth–Average bandwidth of f_o_ peaks; cvint–Coefficient of variation of total intensity; sr–Speech rate; f_o_peakwidth–Average bandwitch of f_o_ peaks.

## Discussion

Gender is socially constructed and undergoes constant change [1]. As a result, expressions and perceptions of masculinities and femininities can vary in accordance with the context of the locutor and interlocutor. As such, this study probed the associations between the perception of gender presentation in the voice and the gender and context of the listener, presenting acoustic measurements that can predict gender presentation in the voice. The study innovated by including the perception of individuals of different genders, including the transgender population, which typically does not participate as judges in the APA.

By way of acoustic analysis of the voices of the speakers, cis and trans women presented median f_o_ values higher than other groups. Voice breathiness also was higher in cis women, non-binary people, and trans and/or travesti women, presented in order of decreasing breathiness (Table 2), potentially having influenced the perception of gender presentation by the judges that used vocal quality acoustic measurements as predictors of gender presentation.

The extracted acoustic measurements of f_o_ and breathiness (Table 2) evidence a gradual increase in breathiness as the gender presentation of the speaker changes. This finding reveals a fluidity in expression and that, for the two aforementioned acoustic measurements, each gender is a point on a continuous scale, ranging from the highest f_o_ and breathiness levels in cis women, moving across trans women, non-binary people, and trans men, to the lowest levels in cis men. Breathiness is indeed an important point to be considered in gender presentation and is an aspect specifically worked on during voice feminization therapy [21].

In the present study, cis men presented higher levels of vocal quality disturbances (as shown by AVQI scores), followed by trans men and non-binary people. Moreover, cis and trans men also had lower f_o_ values (Table 2). The AVQI, a multifactorial measurement to generate a single score, is considered to be one of the most robust objective acoustic measurements of voice quality and severity of dysphonia, providing cutoffs for disturbance levels that do not vary with the gender of the speaker [53, 54].

The linear regression analyses indicated the relationship between judgement of gender presentation in the voice by listeners and the acoustic measurements extracted from the voices of the speakers. This statistical method investigated the linear relationship between APA and acoustic measurements. The predictive analysis of APA provided valuable information that can redirect the currently predominant binary viewpoint of gender presentation in the voice, including in a speech and language pathology clinic setting.

During the evaluation process, it’s common for an analysis of vocal quality to be performed with a higher number of vocal tasks, considering the sustained vowel and connected speech, because vocal behavior is expected to vary by task [55]. In addition, it is worth noting that as the complexity of the task increases, so does the amount of acoustic and communicative information that is made available to influence the judgement of the listener [55]. This relationship was also made evident by the number of acoustic measurements used in APA found in this study, which was lowest in the sustained vowel samples, increased in the connected speech samples, and was highest in the poem recital samples.

In the sustained vowel samples, only the SLP group was found to have acoustic measurements that coincided with their APA, all of which were associated with f_o_ (Table 4). In the same samples, perception of gender presentation by this group diverged from the perceptions of the other two (Fig 5). Speech and language pathologists commonly evaluate vocal quality through sustained vowel samples [55], which would explain this finding. Nonetheless, this group still seems to have considered only the perception of frequency in their evaluations of gender presentation, even though the literature [18, 56, 57] indicates the need to consider additional factors other than those related to the frequency of the voice as important for gender presentation in the voice.

In the evaluation of connected speech (counting numbers), median f_o_ was the most important parameter for the perception of gender in all judge groups. However, judges from the CG group also used HNR to evaluate other cisgender speakers (Table 5) and judges from the TNB group included acoustic measurements of breathiness, such as ABI and CPPs, to evaluate other trans and non-binary speakers (Table 6).

These results may indicate that a listener considers different aspects to identify genders that are different from their own. Additionally, this indicates that judges from the TNB group use acoustic measurements of vocal quality in their evaluations. Interlocutors that share similar contexts and experiences tend to have more effective communication [58], which could explain the difference in evaluation by judges that have a different gender from the speaker.

Cisgender people (SLP and CG groups) tend to consider f_o_ more in judgements of gender presentation in the voice, while trans and non-binary people also react to aspects of vocal quality. The perception of gender presentation by judges in the TNB group were seen to differ from the CG group and, importantly, from the SLP group. Professionals in the area have, in theory, a broader perception of vocal quality and other vocal aspects than even the trans population, something that has become increasingly more common in SLP clinics. The impacts of these findings could lie in the unattainable expectations of femininity or masculinity in the voice, seeing as the visions within the clinic do not converge with those of the clients.

Additionally, it is important to consider how professional biases can impact voice assessment. For instance, linguistic biases can shape SLPs’ attitudes towards clinical scenarios, suggesting that similar biases may occur in the evaluation of voice characteristics related to gender [59]. Similarly, differences in speech accuracy assessment between SLPs and untrained listeners suggest that professionals’ training and experience may either mitigate or amplify biases [60].

These issues may explain why judges from SLP and TNB groups use different acoustic measures to evaluate the voices of transgender and non-binary individuals. Gender perception and the way acoustic measures are utilized may be influenced by personal experiences and implicit biases, underscoring the importance of ongoing training and clinical practice that recognizes and minimizes such biases. A speech and language pathologist must respect the autonomy of a trans person as their client during the construction of the therapeutic process. To do so, they must develop a keen ear with the objective of respecting the goals of vocal performance belonging to that person.

The results from the predictive analysis involving acoustic measurements extracted from the poem recital highlight that there is a difference in how a listener evaluates speakers that play the same gender roles as them compared to other genders. Judges from the CG group evaluated cisgender speakers using fewer acoustic measurements compared to trans and non-binary speakers (Tables 7 and 8). The same occurred for the SLP group which, when evaluating cisgender speakers, utilized only two acoustic measurements; though when they evaluated trans and non-binary speakers, a total of eight acoustic measurements were utilized. The TNB group also seemed to consider more acoustic measurements when evaluating other trans and non-binary speaker; however, differently from the other two judge groups, more acoustic measurements not related to f_o_ were included as predictive of APA. These data indicate that the acoustic cues that each listener utilized to perform APA were different, being possibly related to the different life experiences of these individuals, as well as the type of vocal material they are exposed to in their day-to-day lives and in the media. These data also reinforce the importance of previous experiences when performing Auditory-Perceptual Assessment [55, 61].

In practically all significant regression models, f_o_ was present; even though it is associated with other acoustic measurements, it is still relevant to gender presentation in the voice. However, gender presentation changes as society changes [1] and how femininity and masculinity are perceived can also change over the years.

All judge groups used a larger number of acoustic measurements to judge the speech of trans and non-binary people. In studies with artificial intelligence [62, 63], there is not complete accuracy when judging binary gender presentation in the voice of cisgender speakers; it becomes evident that the less contact a listener has with the gender presentation of the speaker being evaluated, the more cues a listener needs to perform APA. Trans and non-binary people can have a more fluid gender expression, making it more difficult to evaluate them categorically, requiring judges to use more auditory cues to evaluate the gender presentation of these voices.

Perceptions of the human voice have importance in social communication and allow the recognition of various pieces of information about the identity of the speaker [64]. This recognition begins in early childhood [65] and develops to the point at which a listener can recognize speakers by their voice, as well as form ideas about that person’s gender, age, ethnicity, and social status [66]. Nonetheless, this type of recognition depends on the auditory memory and type of vocal sample that the listener is exposed to throughout their life [67]. As such, when judging voices of people with a more fluid gender presentation, listeners can have more difficulty due to their limited or even lack of previous experience with similar vocal models.

A recent study [64] using neuroimaging provided evidence that different cortical regions are involved in the processing of different types of vocal information. As such, the recognition of gender presentation of a speaker though their voice is a higher-level cognitive ability in which linguistic, affective, and identity information are processed in partially segregated cortical pathways. This reinforces that gender presentation is therefore a continuous and not a categorical variable; there is not only one way to be feminine or masculine, but rather many possibilities between two extremes. For the general population, voices that do not fit into predetermined social patterns require more attention for their evaluation.

As such, it would be natural to suggest that trans and non-binary people, as a result of having other life experiences than masculinities and femininities, possibly regard the roles and presentation of gender in a different way than cisgender people. This is confirmed by the divergence in the perception of gender presentation by APA from the speech samples from the TNB group compared to the other two groups (Table 3 and Fig 5).

Specifically regarding the differences found in the perception of SLP judges, these differences in evaluation may reflect in clinical practice. It is known that heterosexual health professionals or those with limited exposure to sexual and gender diversity tend to have more negative biases when working with the LGBTQIAPN+ population, suggesting that such biases may affect how professionals assess vocal characteristics of sexual and gender minorities [68]. It is observed that microaggressions and biases can impact clinical care, which aligns with the idea that SLPs’ training and experience can influence their gender evaluations [69]. Therefore, the difference in the use of acoustic measures between groups may reflect not only a technical approach but also the need for greater sensitivity to professional biases and a better understanding of individual clients’ experiences.

Our findings indicate that personal experiences of professionals in speech and language pathology can hold a more leading role in evaluating gender presentation in the voice. Such experiences can add to their technical knowledge, reinforcing the need to break the paradigm of voices fitting into binary categories.

Although the voice samples of 47 participants were collected in this study, there is still no data in the literature that can allow for sample size calculations. This could result in limiting the translation of these results to the general population. Moreover, the study was carried out with native speakers of Brazilian Portuguese, further limiting generalization capacity since the perception of gender through the voice can be influenced by cultural norms and specific languages.

The absence of conversational speech samples was compensated for by the inclusion of specific tasks that capture relevant nuances in vocal production, thus providing a comprehensive and meaningful approach to achieve the research objectives.

The research aimed to investigate gender perception in the voice, taking into account different groups of judges with a different background and a diverse cohort of speakers. Thus, the adopted methodological choice sought to explore the nuances and complexities present in the auditory-perceptual evaluation of vocal gender in a specific sample, without the need for a traditional control group. The focus was on understanding the dynamics involved in interpreting gender presentation in the voice, considering variables such as the gender identity of the speakers and the experience of the judges, without imposing restrictive conditions that a control group might introduce.

Nonetheless, the experimental design and the results of the present study can offer important insight for the execution of similar investigations with native speakers of other languages and cultures.

## Conclusion

There is an association between the perception of gender presentation in the voice and the gender or context of the listener and a speaker. The study showed that gender and the context of the evaluator influence the perception of gender presentation in the voice. The acoustic measurements and the group of judges also affect perception. Transgender and nonbinary judges diverged to a larger degree from cisgender judges and speech and language pathologists. All judge groups used more acoustic measurements to analyze the speech of trans and nonbinary people. The results indicate the necessity to evaluate gender presentation as a continuous and non-categorical variable. More research is necessary to understand the perception of gender presentation in the voice and its relation to communication, considering the perspective of trans people. The prominent representation of trans people can contribute to a redefinition of gender but requires collective and public political initiatives.
